# Rediscovery of mononuclear phagocyte system blockade for nanoparticle drug delivery

**DOI:** 10.1038/s41467-024-48838-5

**Published:** 2024-05-22

**Authors:** Ivan V. Zelepukin, Konstantin G. Shevchenko, Sergey M. Deyev

**Affiliations:** 1 Department of Medicinal Chemistry, Uppsala University, 751 23 Uppsala, Sweden; 2 Shemyakin-Ovchinnikov Institute of Bioorganic Chemistry of the Russian Academy of Sciences, 117997 Moscow, Russia; 3 Department of Chemistry, Boston University, Boston, MA 02215 USA

**Keywords:** Biomedical materials, Organic molecules in materials science, Nanoscale biophysics, Drug delivery

## Abstract

Rapid uptake of nanoparticles by mononuclear phagocyte system (MPS) significantly hampers their therapeutic efficacy. Temporal MPS blockade is one of the few ways to overcome this barrier – the approach rediscovered many times under different names but never extensively used in clinic. Using meta-analysis of the published data we prove the efficacy of this technique for enhancing particle circulation in blood and their delivery to tumours, describe a century of its evolution and potential combined mechanism behind it. Finally, we discuss future directions of the research focusing on the features essential for successful clinical translation of the method.

## Abstract (web summary)

Temporal blockade of the mononuclear phagocyte system is an approach to enhance the therapeutic efficiency of nanocarrier drug-delivery systems but the broad applicability is hindered by the complexity of optimisation and management of potential side effects. Here, the authors review the development of this technique and show its efficiency using meta-analysis of the published data and discuss essential features for its successful translation to clinic.

## Introduction

Over the past few decades, numerous types of nanoparticles have been studied for disease diagnostics, tumour imaging, and therapy. Through a diverse range of modifications, they recognise and bind specific cellular targets, analyse the microenvironment, and effectively control the release rate of encapsulated drugs^1,2^. Despite these advancements, nanoparticles painstakingly engineered with elaborate functional or architectural features encounter challenges in getting regulatory approval for human use. In fact, it is predominantly liposomal and protein-based formulations that demonstrated clinical success, particularly for the treatment of tumours^3^.

The mononuclear phagocyte system (MPS), previously referred to as the reticuloendothelial system (RES), is a primary barrier that nanoparticles encounter following intravenous administration^4^. The MPS includes resident tissue macrophages in different organs, primarily in liver and spleen, along with blood monocytes, dendritic cells, and their bone marrow progenitors^5^. Upon administration, nanoparticles quickly become coated with serum proteins, which triggers their recognition by MPS cells through a variety of receptors^6^. Thus, many particles exhibit a half-life in the bloodstream of less than several minutes^7^, and less than 1% of the injected dose reaches an intended target tissue^8^. There are several approaches which have proven to be beneficial for prolonging blood circulation time of nanoparticles and enhancing therapeutic efficacy. One encompasses various modification of the nanoparticles to minimise their recognition and uptake by tissue macrophages. This may be achieved through attachment to erythrocytes, camouflaging of the surface with cell membranes or neutrally charged polymers such as polyethylene glycol (PEG)^9–11^. However, the broad applicability of these methods is hindered by the complexity of optimisation and management of potential side effects^12–14^. Another approach relies on the MPS blockade, which refers to the reduction of the system’s endocytosis function in response to macrophage depletion or saturation of the clearance mechanisms. These methods are universal and can be applied to particles of any size and composition, including those with complex architectures. They are also complementary to the other methods for the management of macrophage uptake problem.

In this perspective, we introduce and analyse the evolution of the strategies for the MPS blockade, highlighting the latest innovations in the field. We also address the challenges that need to be overcome for further development of this approach for clinical translation. We propose an integrative mechanism that regulates the blockade, induced by elimination of blocking nanoparticles. Finally, we present a meta-analysis of the current efficiency of this approach for prolongation of blood circulation and improving tumour delivery.

### Evolution of the MPS blockade approaches

**Fig. 1:**
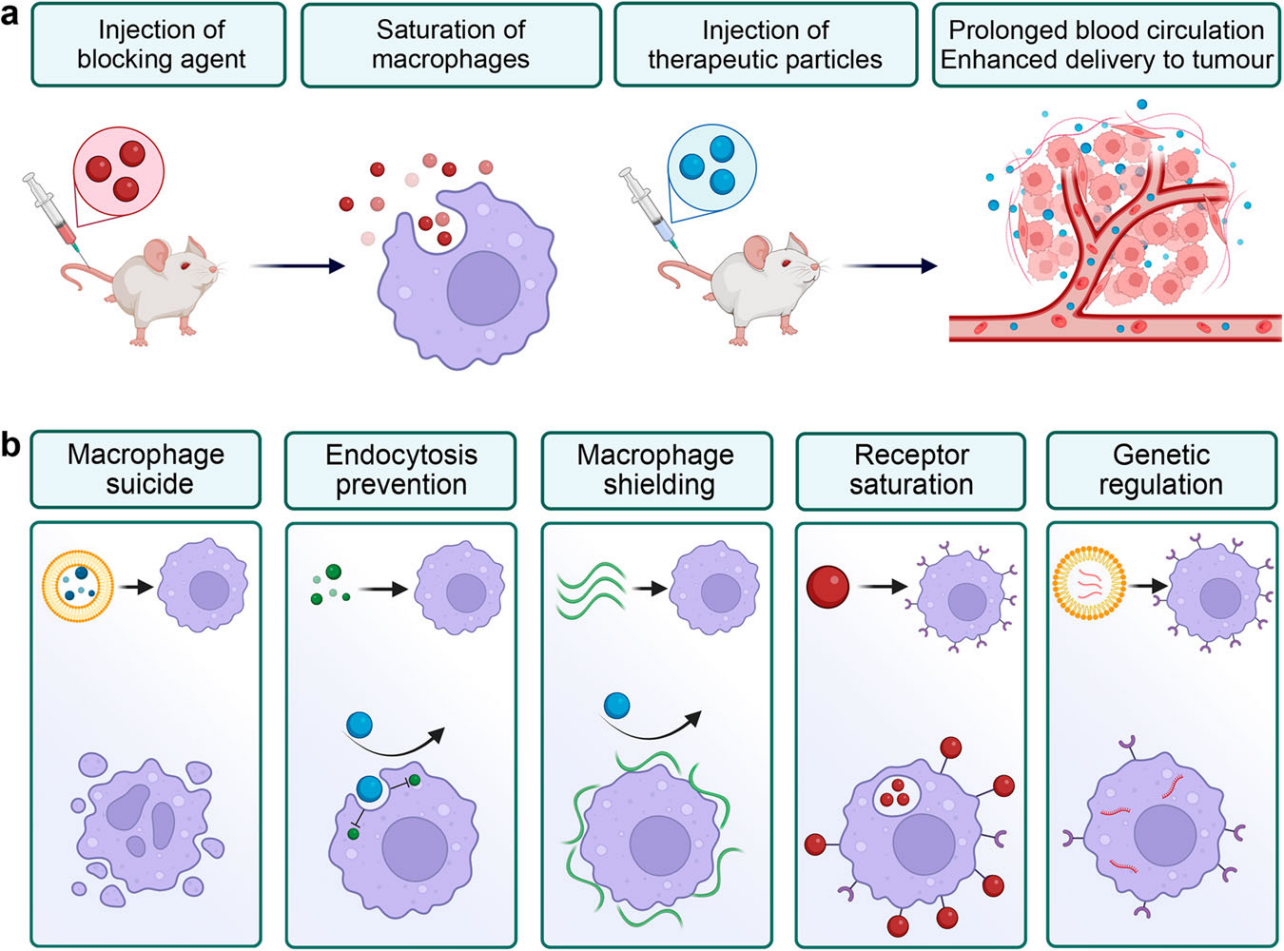
Overview of MPS blockade strategies. **a** General concept of the MPS blockade. Blocking agent (red), when administered to the bloodstream is eliminated by macrophages thereby saturating them. Subsequently administered therapeutics particles (blue) avert macrophage recognition, have longer blood circulation and better accumulation in target tissues. **b** Selected strategies for the MPS attenuation. Left to right: elimination of macrophages with toxic compounds; prevention of endosomal processing by molecular inhibitors; shielding of macrophage surface; saturation of receptors by nanoparticles; genetic downregulation of endocytic receptors. Created with BioRender.com.

The approaches to the MPS blockade, which have been tested so far and which are historically fall under this definition, may be categorised by their impact on the immune system. The MPS depletion eliminates the macrophages until new progeny differentiates from monocytes. This long-term MPS function blockade may be achieved by administration of toxic compounds such as high doses of liposomal clodronate^15^ or gadolinium chloride^16^. A more sparing MPS attenuation method relies on the macrophage pre-treatment with blocking nanoparticles which saturate the uptake pathways and minimise clearance of the subsequently administered tracer nanoagents with therapeutic or diagnostic function (Fig. 1a)^17^. The recent years has given rise to a molecular based strategies that include genetic^18^ and molecular downregulation of endocytosis in MPS cells^19,20^.

From the discovery of the MPS blockade in the early 1920s, the technique has undergone several cycles in which it has come into, and subsequently fall from favour. In the past decade, this strategy was reinvented several times under different names, now referred to as “macrophage priming”, “preconditioning,” “inverse targeting,” “eat me strategy,” and other similar terms^20–23^. Despite employing distinct approaches, all these methods exploit the shared mechanism of the blockade. Furthermore, the designation “RES blockade” is still widely used, although the “reticuloendothelial system” is immunologically an archaic term as it does not include essential tissue phagocytes, such as monocyte-derived macrophages and dendritic cells^24^.

The first papers on the MPS blockade date back to the era of early research on the physiology of the immune system in the 1910s-1920s. At this time, the immunology community aimed to elucidate the functions of tissue macrophages and endothelial cells through their saturation with carbon-based ink, silver and iron oxide colloids^25^. In the 1950s the MPS blockade resurfaced as a key method to study the role of macrophages in physiological response to environmental stress and disease. Administration of colloid carbon, noble metals, gelatine, aggregated albumin, and their combinations temporarily suppressed the phagocytic function and altered the reaction to administration of endotoxins and foreign antigens. The mechanistic basis for this phenomenon has also been established in this period. The MPS blockade was linked to decline in opsonic activity of blood serum, saturation of phagocytic cells, and macrophage damage^26–29^. However, most of the experiments of the era lacked harmonised methodology for nanoparticle fabrication and characterisation. Such properties of the blocking nanoparticles as physical size, surface charge, composition, and colloidal stability, were often described vaguely, which hampered interstudy analysis^26,30^. Nonetheless, these data were foundational for future research in the area.

Despite the diverse range of developed approaches, the MPS blockade has not been used to improve drug delivery to specific tissues until a pioneering study by Proffitt et al. in 1983^17^. They demonstrated enhanced accumulation of radioactively labelled liposomes in tumours upon their administration shortly after the induction of the MPS blockade. The depletion of macrophage function was achieved via administration of amino mannose-modified liposomes that targeted liver macrophages, likely through CD206 mannose-binding receptors^31^. Unlike previous methods, the blocking liposomes were intended to be non-toxic to the liver and spleen, even in high doses^32^. This study has shifted the primary focus of the subsequent research from improving the circulation time of nanoparticles to enhancing their tumour delivery.

The introduction of PEGylation in the next years has garnered a significant interest as a universal method for prolonging drug circulation. Rather than being seen as complimentary approaches, these two strategies were perceived as competitors, with the MPS blockade regarded as less suitable for clinical use^33,34^. Consequently, in the following decades it received little attention until the reinvention in the 2010s^32,35^. A pivotal study by Liu et al. has demonstrated the temporal MPS blockade after administration of liposomes of broad size distribution^32^. The saturation of macrophages improved MRI imaging of the tumour with iron oxide nanoparticles and enhanced the therapeutic efficacy of paclitaxel-loaded vesicles.

This new era is distinguished by several notable features. Firstly, the improved control over physical and chemical properties of nanoparticles has led to better understanding of the fundamental mechanisms underlying the MPS saturation. Secondly, the choice of blocking agents now considers their toxicity profile and suitability for clinical use. Nanoparticles with a short half-life in the bloodstream and a known non-toxic biodegradation pathway have emerged as leading candidates for this role. Finally, potential applications of the MPS blockade have significantly expanded beyond drug delivery. For example, the local blockade of subcapsular sinus macrophages removes the barriers for nanovaccines on their way to lymph nodes, which significantly enhances antibody production^36^. In gene therapy the MPS blockade alters the pattern of gene delivery with chemical and biological vectors. Inhibition of primary uptake by macrophages redirects vectors to other cells and tissues and improves transfection efficacy^21,37,38^. Finally, the MPS blockade delays the clearance of endogenous nanoagents, and by this way maintains their concentration in bloodstream. This approach has been recently employed to increase the sensitivity of liquid biopsy to detect lung metastases in mice through the measurement of circulating nucleosome-bound tumour DNA^39^.

### Strategies to induce the MPS blockade

In this perspective we classify existing approaches to the MPS blockade by the type of a blocker and its mechanism of action (Fig. 1b). One of the most effective ways to attenuate MPS function is to deplete the macrophages with cytotoxic compounds such as bisphosphonate clodronate and diamidine propamidine in liposomal formulations^15^. Both compounds, upon administration in a free form, demonstrate moderate toxicity and rapid excretion by kidneys. However, in liposomal form they effectively reduce the number of Kupffer cells in liver as well as macrophages in red pulp and marginal zone of spleen^40^. The local administration of liposomal clodronate or diamidine propamidine depletes the resident macrophages at the site of the injection. This effect has been demonstrated for subcapsular sinus macrophages in lymph nodes^36^, tissue macrophages in the peritoneal cavity^41^ and perivascular phagocytes of central nervous system^42^. Upon endocytosis the liposomes become exposed to phospholipases in lysosomes and release a toxic compound – first, in endosome and, subsequently, into cytoplasm. There these compounds inhibit mitochondrial ADP/ATP translocase and, in high concentrations, induce apoptosis^15,40^. The chlodronate-loaded liposomes primarily target the same cells which interact first with nanoparticles. For instance, the population of Kupffer cells is decreased by 90% two days after such treatment, thereby, enhancing the tumour delivery of the nanoparticles by up to 150 times^43^. Molecular clodronate can be metabolised and rapidly cleared from the body, so, monocyte-derived macrophages may slowly repopulate their niches in a period ranging from several weeks^43^ to months^44^. Due to the long-term effects of clodronate on innate immunity, this approach has a relatively low potential for translation in the clinic. Nonetheless, this method became a sustained instrument in fundamental research of macrophage function.

Another macrophage-depleting agent is gadolinium chloride which suppresses phagocytosis in Kupffer cells by inhibiting Ca^2+^ transport through cell membrane^45^. Such blockade decreases the liver uptake of nanoparticles, which improved subsequent tumour imaging with long-circulating quantum dots targeted to epidermal growth factor receptor (EGFR)^46^. Recently it has been used in preclinical rat model to induce immune tolerance and to suppress acute rejection of liver allografts^47^. However, high dose of this agent eliminates significant parts of Kupffer cells and spleen macrophages similarly to liposomal clodronate^16^. Other toxicity issues include a pronounced increase in proinflammatory cytokine release by macrophages and cardiac toxicity, since calcium current has a pivotal role in cardiac cells functioning^48,49^.

Several other low molecular drugs that impact endosome formation and trafficking have also been evaluated for induction of the MPS blockade. Esomeprazole, a clinically approved proton-pump inhibitor, blocks V-ATPase and alters lysosomal trafficking in tissue macrophages. By hampering the MPS uptake of therapeutic nanoparticles it increases their tumour delivery by 1.8-fold^50^. Another clinically approved molecule that may be used for the MPS blockade is chloroquine, the established antimalarial agent. Its mechanism of action is based on inhibiting endocytosis through the reduction of phosphatidylinositol binding clathrin assembly protein (PICALM) expression^20^. Additionally, chloroquine prevents lysosomal acidification and hinders fusion of endocytic vesicles^51^. Administration of chloroquine in a clinically relevant dosage reduces the liver uptake of liposomes by 28.5% without any significant toxic effects to Kupffer cells. This is accompanied by two-fold increase in liposomal delivery to MDA-MB-231 breast tumours in mice^20^. A third notable compound is methyl palmitate. It transiently alters the architecture of the cellular microtubule network, which in turn hampers nanoparticle internalisation^52^.

A different approach to induce the MPS blockade is saturation of scavenger receptors by various polymers. For instance, 500-kDa dextran-sulphate and fucoidan, natural seaweed-derived polymer, were shown to block the uptake of liposomes by macrophages and to improve the consequent MRI-contrasting by colloid iron oxides^53,54^. Both polymers bind scavenger, Fc, and C3 receptors, and induce significant redistribution of nanoparticles from liver to other organs^55^. However, dextran-sulphate failed in clinical trials due to its systemic toxicity, which manifested as severe thrombocytopenia and alopecia^56^. Several other polymer antagonists of scavenger receptors such as polylysine peptides^57^ and polyinosinic acid^58^ have been studied recently. Interestingly, they are effective in vivo even in comparatively low doses which may be explained by their binding to serum proteins. This binding may lead to in situ generation of aggregated albumin nanoparticles which become cleared by resident macrophages^57^. Also, some of the branched polymers are capable of long-term shielding of cell surface hampering the interaction with other agents. For instance, biarmed polyethylene glycol covers sinusoidal endothelial walls and alters biodistribution of adeno-associated viruses and polyplex micelles^59^.

Preconditioning of macrophages by administration of harmless blocking particles has been historically the most popular strategy to induce the MPS blockade. Administration of liposomes temporarily saturates macrophage receptors and depletes serum opsonins^32,60^. The blocking effect in most cases is temporal and lasts from several hours to days^61,62^. The application of the nanoparticles with known safe biodegradation pathways such as ferrihydrite, silicon and polymeric ones is another way to ensure the low long-term toxicity of the blockade^63–66^. Interestingly, induction of the blockade may also be an off-label application for some of the regulatory approved formulations. They include intralipid, a lipid emulsion, authorised for parenteral nutrition and recognised as safe for multiple administrations. In rats its systemic administration decreased liver uptake of magnetic iron oxide nanoparticles two-fold and therefore increased the efficiency of the magnetic nanoparticles delivery to blood monocytes^67^. Moreover, intralipid pre-treatment reduced acute splenic toxicity of several nanotherapeutics through alteration of their pharmacokinetic profile^68^.

In the same manner the macrophage saturation may be achieved through enhancement of elimination of endogenous agents such as aged red blood cells (RBCs). In humans about 1% of RBCs are recycled daily and part of them is cleared by Kupffer cells. Intensification of this process by systemic administration of anti-RBC antibodies has been recently shown to induce the MPS blockade^69^. This method enhanced tumour delivery of nanoparticles up to 23-fold and significantly improved the therapeutic efficacy of liposomal doxorubicin. Other similar strategies suggested the use of antilymphocyte serum and polyclonal anti-RBC antibodies^69,70^. The toxicity and efficacy of this technique might be further optimised by administration of antibody-pretreated cells. Alternatively, macrophages targeting anti-Fc-receptor antibodies may be employed to inhibit endocytosis^71^, but the efficacy of this method for potentiation of nanoparticles has not been studied.

Several recent studies revised the macrophage overloading mechanism of the MPS blockade. The first method relied on downregulation of phagocytosis through the activation of signal regulatory protein alpha (SIRPα) by CD47-mimicking peptides. Peptide modified liposomes bound SIRPα on the surface of Kupffer cells and liver sinusoidal endothelial cells but escaped internalisation. Instead, they formed a long-term shield, which prevented uptake of the administered therapeutic nanoparticles and significantly reduced their clearance^72^. Another strategy employed genetic engineering to repress endocytosis in tissue macrophages and to reroute the microRNA delivery vehicles to target cells. The macrophages were primed with exosomes bearing blocking siRNA against clathrin heavy chain 1. It downregulated clathrin-mediated endocytosis, a primary mechanism of exosome elimination. Next, therapeutic microRNA miR-21a-5p was administered using the same type of vehicles. Functional depletion of MPS enhanced the uptake of therapeutic exosomes in heart tissue and improved treatment of doxorubicin-induced cardiotoxicity in animals^18^.

### Combined mechanism of the MPS blockade by nanoparticles

**Fig. 2:**
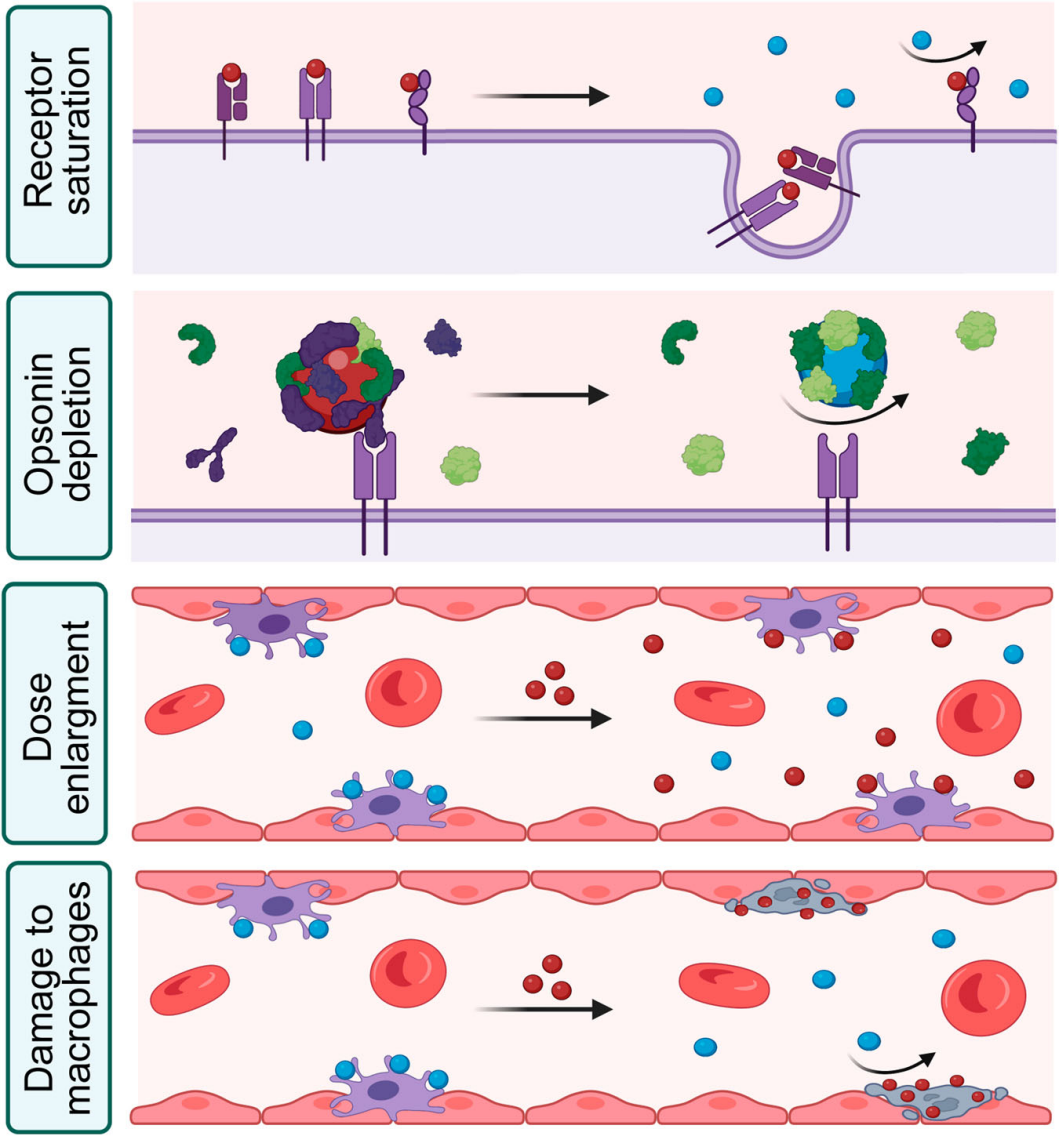
Different aspects of the combined mechanism of MPS blockade by nanoparticles. Top-down: i) Blocking particles (red) saturate receptors and induce their internalisation. After that therapeutic nanoparticles (blue) can’t bind receptors on the surface of macrophages. ii) Blocking particles bind and deplete opsonins (violet proteins), which hampers interaction of therapeutic particles with receptors. iii) High dose of circulating blocking particles saturate the macrophage uptake rate and therapeutic particles are unlikely bind the cell surface. iv) Following the endocytosis of blocking particles, part of macrophages can be damaged and eliminated. Created with BioRender.com.

The MPS blockade is efficiently achieved by various strategies, which suggests a complex set of factors that govern this process (Fig. 2). Generally, it is mediated through the saturation or overload of nanoparticle clearance by tissue resident macrophages. However, the precise underlying mechanism remains unclear.

Nanoparticle elimination starts with opsonisation, i.e., their coating with plasma proteins such as immunoglobulins, complement factors, fibrinogen, and other proteins which enhance phagocytosis of the foreign materials by macrophages. Macrophages express a plethora of surface receptors that recognise opsonised nanoparticles, such as Fc, complement and scavenger receptors^4^. Phagocytosis appears to be a predominant mechanism of nanoparticle internalisation by macrophages in vivo^73^, although caveolin- and clathrin-dependent endocytosis are also involved. Phagocytosis is a typical uptake route for nanoparticles larger 100 nm, but it may also eliminate the clusters of the smaller particles, as confirmed by electron microscopy^74^.

The most evident mechanism of the MPS blockade is the saturation of certain opsonin receptors. Nanoparticles usually occupy membrane receptors for dozens of minutes before they become internalised^74^. That temporarily reduces the number of available receptors on macrophage surface. Most of the known opsonin receptors are involved in the MPS blockade. Exosomes coated with cationic mannan saturate macrophage CD206 mannose receptors, which suggests regulation of this process by specific receptors-ligand interactions^23^. On the other hand, Fc-receptor mediated phagocytosis may be saturated by sensitised red blood cells which reduced the uptake of subsequently presented liposomes^69^. Scavenger receptors class A are involved in dextran-coated particle clearance^75^, while blockade with this polymer alone also temporally depletes C3 and Fc receptors^55^. However, the knockout model in RAW 264.7 macrophages controversially suggested minimal input of scavenger receptors B1 in the liposome-induced blockade, although they are known to participate in recognition of the liposomes^32^. The current understanding of precise mechanisms by which opsonic receptors are involved in the MPS blockade is fragmentary and requires further studies in knockout cell and animal models.

Depletion of serum opsonins is another important part of the combined mechanism of blockade. Administration of liposomes in high doses, typical for the MPS blockade, significantly decreases the amount of proteins bound per nanoparticle^76^. It is likely possible that the pool of opsonins necessary for clearance of therapeutic particles can be physically exhausted^76^. This notion is supported by the fact that in some cases blocking nanoparticles with a coating different from the tracer did not prolong circulation of tracer particles, even when administered at high dosage^77^. In another study, Ni^2+^-chelated liposomes induced 2.3-fold prolongation of iron oxide circulation than unmodified liposomes, which may be explained by similarity between plasma opsonins that have affinity for nickel and iron oxides^78^. Concurringly to the opsonin exhaustion concept, incubation of tracer nanoparticles with a fresh serum prior to their administration ameliorates the effect of the blockade and significantly accelerated their elimination^28,77^. The recovery time also partially correlates with a restoration of opsonin activity in blood^28^. The architecture and shape of nanoparticles directly impact the composition of the protein corona and their interaction with immune cells. We expect the future research will shed light on the precise mechanisms underlying this phenomenon and will empower the rational design of blocking nanoparticles.

The recent findings have added one more dimension to the complexity of cell-nanoparticle interactions that govern the blockade. The presence of blocking particles in bloodstream may affect the efficacy of the blockade per se as the MPS has a specific maximum uptake rate of nanoparticles^79^. Blocking nanoparticles are typically administered in a high dose that saturates uptake rate by macrophages and results in their hours-long circulation in bloodstream. When therapeutic particles are introduced before the complete clearance of the blocking ones, both agents start to compete for opsonins and endocytic receptors at macrophages.

Apparently, saturating uptake rate is likely more important for the MPS blockade than saturating macrophage capacity. Using real-time intravital microscopy Ouyang et al. have shown that macrophages are saturated shortly after injection of blocking gold nanoparticles. In this study blocking particles occupied only a relatively minor volume in Kupffer cells and uptake capacity was not saturated. The elimination of nanoparticles processed through receptor-mediated phagocytosis and the blocking particles swiftly overload the binding sites on macrophages. It prevents further binding and internalization of therapeutic nanoparticles by these cells^80^. Another study of MPS blockade with sensitised RBCs show that maximum inhibition of phagocytosis was achieved in 12 h after administration of anti-RBC antibodies, although haematocrit was reduced only by 0.8%^69^. The MPS blockade alleviated in 5 days, while the total RBC concentration decreased by 5%. It confirms that rate of RBC clearance was more crucial for the success of MPS blockade than the total quantity of eliminated erythrocytes. Moreover, a certain threshold was determined for the saturation of uptake rate, equal to 1 trillion particles per mice^80^. The dosage of nanoparticles, used to induce the blockade is usually higher than this threshold, and therapeutic particles are typically administrated shortly afterwards. It means the blocking agent is still partially present in the bloodstream by that time^79,81^. In some cases, the duration of the blockade correlates with the amount of remaining blocking material in the blood^26,82^. This insight was used to improve liposomal drug delivery, where the uptake rate was saturated by simultaneous administration of therapeutic liposomes with a high concomitant dose of empty particles^80^. Pharmacokinetics analysis of the blocking nanoparticles is crucial for better understanding of the mechanisms underlying the MPS blockade.

Finally, high doses of nanoparticles may damage macrophages or induce the cell death. For instance, the administration of colloid carbon results in two phases of phagocytosis paralysis. While the initial paralysis may be explained by the factors mentioned above, the delayed repression occurs even after complete clearance of blocking particles from the bloodstream^27^. Interestingly, this phenomenon has not been observed for the blockade with biogenic particles of aggregated albumin. In other studies toxic and radioactive particles also induced long-term damage to Kupffer cells and decreased the MPS uptake^74,83^. It is worth mentioning that tissue macrophages are replenished from blood monocytes and their repopulation takes from several days to a weeks after a blockade with toxic particles^43,84^.

Nevertheless, the impact of macrophage damage on the blockade phenomenon became diminished in recent studies. Currently, the MPS blockade is generally induced by materials with safe biodegradation pathways. Histological and biochemical studies support the lack of liver and spleen toxicity for many tested materials^32,69^. Nevertheless, since liver and spleen macrophages do suffer from the blocking particles, toxicity analysis for these tissues should be carried on for safe translation of the MPS blockade to the clinic.

### Meta-analysis of nanoparticle induced MPS blockade

The MPS blockade has gone through several iterations before it reached its current state of implementation for therapeutic purposes. Similar in their general concept, the experiments differed in methodology which adjusted to the technological advances of time. Here we attempt to evaluate whether current technology benefited the efficiency of this approach through bringing the data from different studies to a common standard and its further comparative analysis.

For this goal we identified and reviewed 153 studies related to the MPS blockade through a comprehensive search in Google Scholar and PubMed databases. Next, we performed the meta-analysis of the studies which employed nanoparticles in high doses or cells to saturate macrophage uptake rate and capacity. As blocking agents, they have similar mechanism of action. The number of papers which dealt with other saturation approaches was too small for a sufficient analysis, while the studies which employed clodronate liposomes were excluded because of the toxicity of this approach. For every paper, we deduced the parameters of the administered blocking and tracer particles. We extracted the relevant data on blood pharmacokinetics, biodistribution, and tumour delivery efficiency of tracer particles where applicable. For the comparative analysis of blood pharmacokinetics, we generally used half-life time (t_1/2_). The area under curve (AUC) parameter was analysed if the kinetics curve significantly differed from monoexponential behaviour. Tumour delivery efficacy was also analysed by AUC if several time points were reported. The obtained numbers were then normalised to the related values for pharmacokinetics of tracer particles in the absence of the MPS blockade. We plotted the results as Tukey-type box plots, describing median and 25-75% percentiles. We used median values for comparison as it less dependent on the variability of data than mean value. Statistical difference was analysed by ANOVA with Tukey’s post-hoc comparison. The detailed description for the methods of data collection, standardisation, and statistical analysis can be found in Supplementary Information. The raw data are presented in Supplementary Datasets 1, 2; normality tests and data transformation are presented in Supplementary Table 1 and Supplementary Figs. 1–5; descriptive statistics and statistical analysis data are presented in Supplementary Tables 2–5.

**Fig. 3:**
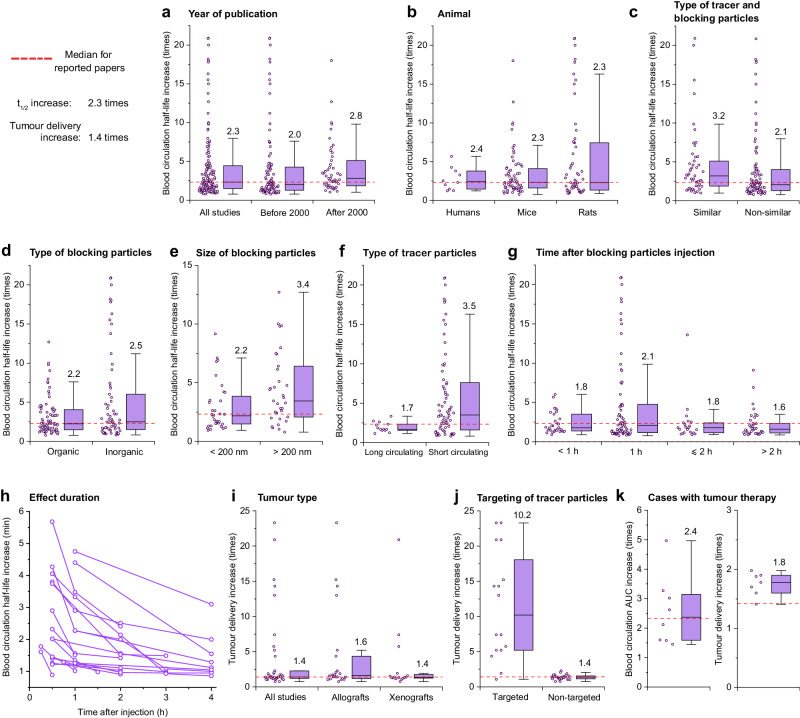
Analysis of MPS blockade efficiency. **a**–**g** Increase in half-life time (t_1/2_) of tracer nanoparticles after the induction of the MPS blockade. Red line indicates median efficiency derived from all data sets and equals to 2.3-fold increase. **h** Decrease of the MPS blockade efficiency over time after the administration of blocking particles. Each connected line shows a separate case of MPS blockade from the Supplementary Dataset 1. **i**, **j** Increase in the efficiency of tumour delivery for tracer nanoparticles after the induction of the MPS blockade. Red line indicates median efficiency derived from all data sets and equals to 1.4-fold increase. **k** Analysis of increase in blood circulation by AUC (left) and tumour delivery (right) in datasets, where tumour treatment was demonstrated. The boxes represent median, the 25th to 75th percentiles, and the whiskers show 1.5 interquartile range. Analysed data points are plotted left to the boxplots. Median values are reported on the graphs.

The increase in blood circulation for tracer particles after the MPS blockade has been reported in 144 cases with the median increase in 2.3 times compared to the control (Fig. 3a). Importantly, efficiency of the MPS blockade was equal in mice, rats, and humans (*p* = 0.75; Fig. 3b). In addition, it did not depend on the immune status of the animal or the presence of a tumour (*p* = 0.197, Supplementary Fig. 6). Based on these findings we discarded the data breakdown by species from further analysis.

The recent spike in interest in the MPS blockade has coincided with the advancement in its efficacy. In the last two decades, the reported efficacy of the blockade has improved by 40% compared to the previous years. The median prolongation of t_1/2_ increased from 2.0 times to 2.8 times (*p* = 0.027), while the variability of the data did not change (Fig. 3a). We have also deduced other trends from these data. Below we describe how the properties of nanoparticles, administration regime and the host factors may contribute to the efficiency of the MPS blockade in terms of t_1/2_ prolongation.

### Influence of blocking and tracer nanoparticle nature

Firstly, the increase in t_1/2_ depends on the type of blocking nanoparticles. The MPS blockade is more efficient if the blocker and tracer particles have similar composition (*p* = 0.019; Fig. 3c). For example, blocking liposomes better prolong the circulation of therapeutic liposomes, than of other polymeric or inorganic nanoparticles. The reported median t_1/2_ increases 1.5-fold higher for similar pairs of tracer and blocking particles, than for different ones. This effect may be explained by resemblance in composition of protein corona and clearance mechanisms of the nanoparticles of the same nature. In this case the blocking agents eliminate specific opsonins and better saturate specific receptors, required for endocytosis of the tracer particles. Interestingly, the nature of the blocking particles in general has a negligible effect on the efficacy of the blockade (*p* = 0.418, Fig. 3d). The median increases in t_1/2_ induced by organic (liposomes, polymeric particles, cells, and aggregated proteins) and inorganic materials were 2.2 and 2.5 times, respectively (Fig. 3d). This observation suggests that arbitrary agents introduced to macrophages may induce a non-specific blockade. It also speaks in favour of more extensive use of biocompatible organic nanoparticles for the MPS blockade.

Secondly, the efficiency of the blockade depends on the size of blocking particles (Fig. 3e). We found that particles > 200 nm in diameter induce a more pronounced blockade compared to the smaller ones (*p* = 0.028) with a median t_1/2_ increase of 3.4 and 2.2 times, respectively. This finding may be justified by a more intensive interaction with Kupffer cells of a larger particles as they are unable pass through the endothelial fenestrae to the Disse space^75^. Also, they exhibit shorter half-life in bloodstream and faster occupation of the binding sites on macrophage surface^7^. Several studies reported stronger blockade by large 1-µm particles compared to 100-nm ones, even if they were administered in two orders smaller molar dose^62,85^.

Thirdly, the MPS blockade is more efficient for tracer particles which initially exhibit a shorter half-life in a bloodstream (here, t_1/2_ < 30 min). Median efficiencies of the MPS blockade were 1.8 and 3.5 for long-circulating and short-circulating tracer particles, respectively (Fig. 3f, *p* = 0.035). Interestingly, the variability of the data significantly changes for short-circulating particles - all the maximum level outliers on the graph come from this group. A good example may be drawn from the recent paper on MPS priming with antibody-labeled RBCs. The authors demonstrated impressive 32.1-fold increase in circulation of the tracer nanoparticles with the initial half-life of only 31 seconds^69^. This observation suggests that the primary objective of the blockade should be delivery improvement for the particles that are swiftly recognised by the MPS. However, it can undoubtedly supplement the other methods that enhance particle circulation.

The efficacy of the blockade is influenced by other properties of the particles as well. However, many papers lack adequate characterisation of blocking agents’ composition, and the meta-analysis is not currently feasible. Nonetheless, it is important to highlight these properties to promote the rational design of the blocking particles in the future.

The stiffness of nanoparticles plays an important role in their interaction with macrophages. A recent study revealed that stiff nanogels effectively induce MPS blockade, while the soft ones evade macrophages and tend to accumulate in tumours^65^. These findings challenge the prevailing use of liposomes and other soft organic particles for the MPS blockade. Particles that were previously considered ineffective due to the rapid clearance might be reevaluated as potential supplementary therapy for macrophage saturation.

The data on the relation between ζ-potential of nanoparticles and the MPS blockade remain contradictory. For instance, positively charged liposomes induce a stronger blockade compared to negatively charged or neutral ones and afford enhanced delivery of gold nanorods to glioma tumours^35^. Another study shows that amino-modified liposomes are more effective than neutral ones^17^. In contrast, negatively charged silicon oxide nanoparticles show higher blockade efficiency compared to the positively charged ones^85^. The influence of ζ-potential on the blockade may be indirect, mediated through such factors as the composition of the protein corona, quantity of adsorbed opsonins, particle aggregational stability in blood serum, and eventually the speed of their recognition and uptake by macrophages.

### Influence of the regimen of nanoparticles administration

The blood bioavailability of therapeutic nanoparticles after the MPS blockade has likely dose dependent character^27,72^. The increase in the dose of blocking carbon particles does not significantly affect the saturation of macrophages but significantly prolongs the duration of the observed effect from 0.5 to 13 h^27^. This data is concurrent with a study in human patients which investigated the blockade induced by gelatine particles in different dosages^81^. This long-lasting effect may be attributed to the prolonged circulation of the blocking nanoparticles administered in high concentrations. They continue to re-saturate the recycled receptors and decrease the uptake rate of tracer nanoparticles by diluting them. Another study analysed the efficacy of the MPS blockade at the moment of the complete clearance of blocking nanoparticles from the bloodstream. It revealed a gradual rise of macrophage saturation with the dose increase^85^. Furthermore, at low doses below 25 mg/kg the silica nanoparticles do not induce MPS blockade after clearance^85^.

In meta-analysis, we evaluated the influence of the time gap between administration of blocking and tracer particles on the efficacy of the blockade. In most studies the MPS blockade was observed shortly after introduction of blocking particles, but the most pronounced effect was achieved one hour after that. Importantly, it confirms the necessity of an interval between administrations of blocking and tracer particles to perform opsonisation of blocking particles and binding them with macrophage receptors. Concurrently with the proposed combined mechanism of the blockade, this period should be short enough to ensure the saturation of the macrophage uptake rate by the blocking particles circulated in the bloodstream. Moreover, at short time intervals a part of endocytic receptors will not be recycled after the uptake^86^, further attenuating nanoparticle elimination rate.

The administration of blocking nanoparticles tends to induce a very short-lasting effect when compared to other approaches. After the reaching the maximal effectivity, the blockade steadily decreases over time. Figure 3h shows the related data from the cases, which report the t_1/2_ increase at several time points after the administration of blocking particles. In most studies the MPS cells restore their function within several days. However, sometimes the effect of the blockade can completely disappear in the first 4 h after the induction (Fig. 3h). A short-term effect may be explained by assuming that nanoparticles temporarily block macrophages activity. The restoration of endocytic function in this case requires only the recovery of cell surface receptors but not the replenishment of macrophage pool. In this connection nanoparticle-based blockade appears to be much safer than it might be perceived. It has been considered a general knowledge that the decrease in macrophage activity may hamper the clearance of bacterial pathogens and cell debris. However, our analysis shows that the blockade with nanoparticles has short duration and typically resolves within hours.

### Influence of host factors

The uptake rate of nanoparticles and the efficiency of the MPS blockade may be influenced by numerous factors, including differences in biology between the model species. The dose required for the induction of the blockade, as well as the corresponding toxicity of the method, may vary greatly depending on the host’s condition. Nevertheless, the MPS blockade is a universal phenomenon observed across different animal classes and both in healthy and diseased ones (Supplementary Dataset 1).

Various species have differences in the quantity of macrophages and their accessibility to nanoparticles. The elimination of blocking nanoparticles is primarily governed by their interaction with liver and spleen macrophages. The architecture of the liver, with macrophages oriented towards the inner surface of sinusoids, is very similar across different animals^87^. At the same time the architecture of the spleen varies between humans and rodents^88^. Furthermore, species differ in macrophage density within tissues. For example, a mouse liver contains 10^7^ macrophages per gram of tissue on average, but this value can vary 2-3-fold in different models^89^. For now only one paper reports the MPS blockade induced in different species within one study, demonstrating virtually the same efficiency for mice and rats at an equal blocking dose^69^. To evaluate the interspecies differences on meta-analysis results we reanalysed the data for mouse and rat animal models separately. In this re-analysis we included only those parameters, where statistically significant differences were observed in the general dataset, i.e. similarity between composition of blocking and therapeutic nanoparticles (Supplementary Fig. 7a), size of blocking nanoparticles (Supplementary Fig. 7b), circulation time of therapeutic nanoparticles (Supplementary Fig. 7c). Importantly, the observed dependencies maintained unchanged in these two animal models, which do have different microcirculatory architecture of spleen.

Next, the quantity and the phagocytic activity of macrophages may be altered in a disease state. For example, the biodistribution of the nanoparticles among the MPS organs is altered in the patients with chronic hepatic diseases^90^. Also, the clearance capacity of the MPS exhibits the age-related decrease in humans by ~15% between 30 and 80 years of age^91^. Thus, the regimen of the MPS blockade induction should be altered accordingly.

Another important parameter, which can influence MPS blockade is the phenotype and polarisation of liver macrophages. The liver contains populations of resident Kupffer cells and bone marrow-derived recruited macrophages. They have similar functions but differ in the activity towards nanoparticle processing. Particularly, monocyte-derived macrophages have reduced expression of scavenger MARCO receptors, that participate in the uptake of nanoparticles and bacteria^92^. Polarisation of the macrophages to M1 or M2 phenotypes may also impact the uptake by altering the expression of surface receptors. The faster clearance was demonstrated in Th2-immune mouse strains compared to Th1-immune ones^93^. Also, the increase in M2-like macrophages in the liver and spleen due to a tumour burden enhances clearance of nanoparticles from the bloodstream^94^. The MPS blockade in mice, bearing melanoma B16-F1 tumours was more effective than in naïve animals^85^. The inverse dependence was observed in mice with EMT6/P adenocarcinoma^85^, which stresses the importance of the MPS blockade evaluation in tumour-bearing animals. Our meta-analysis didn’t reveal any statistically significant differences between animals with and without tumours (Supplementary Fig. 6, Supplementary Table 2), possibly due to low number of available studies for comparison.

Summing up, the host condition may influence the efficiency of the MPS blockade and requires further investigation to evaluate general dependences. Assessing the quantity of macrophages and their specific phenotypes in different animals, as well as during disease, is crucial for optimisation of the technique.

### Tumour delivery and therapy after the MPS blockade

The primary therapeutic target of the MPS blockade is enhancement of drug delivery to tumours and boosting the therapeutic effect of nanoparticle-based formulations. The MPS blockade increases nanoparticle delivery to tumours 1.4 times in median compared to the control values (Fig. 3i). Most cases (38 out of 39) report the MPS blockade in mouse models and 1 case shows nanoparticle delivery to tumours in rats. The median increase was slightly higher for allograft (1.6-fold) than for human xenografts tumour models (1.4-fold). However, this difference is not statistically significant (p = 0.477). The efficiency of the blockade significantly increases for targeted nanoparticles when compared to the non-targeted ones (Fig. 3j). The medians are 10.2- and 1.4-fold corresponding increase in their delivery. In the analysed cases, the authors primarily used for targeting the application of external magnetic fields or functionalisation of nanoparticles with biomolecules capable of receptor recognition on tumour cell surface. In certain cases, the absence of active targeting leads to a nearly complete absence of particle accumulation in the tumour, even after the induction of the MPS blockade^69,78^. It highlights the importance of combining this approach with other methods that enhance nanoparticle retention in tumour and facilitate their permeation across the extracellular matrix and cell membrane.

While we have observed a moderate increase in the efficiency of the MPS blockade in the last two decades, it remains unknown whether these values are sufficient for effective tumour therapy. To address this question, we analysed all the cases in which the MPS blockade demonstrated therapeutic efficiency, i.e., slowing down of the tumour growth or prolonging animal survival. These 13 cases include the blockade of MPS function with nanoparticles, red blood cells and small molecules, as summarised in Supplementary Dataset 2.

Figure 3k illustrates that median prolongation in blood circulation of therapeutic particles is 2.4 times, ranging from 1.5 to 5.0 times. The tumour delivery of therapeutic particles increases 1.8 times after the MPS blockade, ranging from 1.6 to 2.0 times. When compared to these medians 51% of all analysed cases showed higher values for prolongation of tracer nanoparticle circulation (Fig. 3a). At the same time only 34% of the papers reported better efficiency for tumour delivery (Fig. 3i), compared to these medians. This fact demonstrate that current approaches already permit increase in blood circulation of nanoparticles sufficient for effective anti-cancer therapy. At the same time the low tumour delivery is a more complex problem that requires surpassing a number of barriers to retain nanoparticles in the tissue. This problem can be partially resolved by employing active targeting approaches (Fig. 3j)^4^, or rapid drug unloading in the tumour-surrounding microenvironment^95^. Another important aspect is the influence of blocking particles on penetration of the tumour tissue by the therapeutic compounds. It is commonly considered that nanoparticles can accumulate in tumours via leaky vasculature due to enhanced permeability and retention (EPR) effect^96^ or using active transendothelial processes of transport^97^. Blocking nanoparticles can interact with endothelial cells and dramatically affect transendothelial pathway of therapeutic particle accumulation. This aspect of MPS blockade requires further investigation.

Note that MPS blockade almost has not been applied for tumour therapy despite early recognition. The first study by Profitt et al. in 1983 demonstrated improved tumour delivery after the liposomal MPS blockade^17^, while only in 2015 the same type of blockade was applied for treatment of prostate cancer^32^. Recent achievements have allowed to take the first steps in cancer therapy. They include a 40% improvement in the efficiency of the blockade, the development of new classes of biocompatible blockers, and the progress in tumour targeting. We speculate these early results will drive the progress in the area and will bring it closer to clinical applications.

### Changes in nanoparticle biodistribution after MPS blockade

**Fig. 4:**
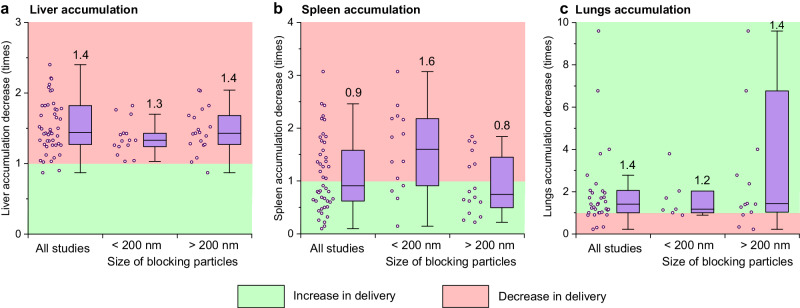
Spillover effect of the MPS blockade. Analysis of the changes in tracer nanoparticle accumulation in liver **a**, spleen **b** and lungs **c** after the induction of the MPS blockade with blocking nanoparticles of different sizes. Green and red zones correspond to the increase and decrease of tracer particle delivery, respectively. The boxes represent median, the 25th to 75th percentiles and the whiskers show 1.5 interquartile range. Analysed data points are plotted left to the boxplots. Median values are reported on the graphs.

The discussion of the biodistribution of tracer particles after the MPS blockade would be incomplete without mentioning of the “spillover effect” (Fig. 4), i.e. redistribution of nanoparticles to other macrophage-rich tissues, following the blockade of the initial MPS barriers^98^.

Upon administration the blocking agent primarily saturates the most accessible cells of the MPS, such as Kupffer cells in liver. This fact may be explained both by their absolute number and by their anatomical location on the surface of hepatic sinusoids where blood flow is slowed down^99^. Therefore, the MPS blockade facilitates transport of therapeutic nanoparticles from liver to other macrophage-rich organs such as spleen and bone marrow. Spillover effect is observed for different blocking agents, including nanoparticles, polymers, cells, and molecular agents such as liposomal clodronate and gadolinium chloride^43,64,69,100^.

The spillover effect may be observed even within the same organ. Depletion of Kupffer cells with liposomal clodronate affects macrophages close to the portal triads at greater extent than the ones which are close to the central vein^43^. In spleen, more accessible macrophages located in marginal zone and red pulp are blocked more effectively than the ones in white pulp^101^.

The meta-analysis supports the predominance of spillover effect for the MPS blockade with nanoparticles, characterised by diminished liver uptake alongside augmented accumulation in the spleen (Fig. 4a, b, raw data in Supplementary Dataset 1). Importantly, we observed dependence of this effect on the size of the blocking particles. Blocking particles larger the 200-nm threshold cause a median 1.3-fold increase in therapeutic particle accumulation in the spleen, while the smaller ones, conversely, provoke a 1.6-fold reduction of the uptake in this organ. Importantly, in both cases the liver uptake of therapeutic particles decreased in median at similar level of 1.3 and 1.4 times for small and large blocking particles, respectively. Spillover effect is likely dependent on other properties of blocking particles, such as shape, ζ-potential, surface coating, etc. It explains why in some cases administration of liposomes or lipid emulsions of broad size distribution did not induce particle redistribution from the liver to spleen^32,102^.

We found no evidence which supports dependence between the size of blocking nanoparticles and the redistribution of the tracer ones to non-MPS tissues. While liver and spleen are macrophage-rich organs, lungs almost have no macrophages oriented to blood vessels. Figure 4c shows that after the MPS blockade, more therapeutic particles accumulate in lungs and the delivery has not been significantly influenced by the size of blocking particles. The increase in particle accumulation in lungs was comparable to the one in tumours (Fig. 3i). That supports the notion that the observed enhancement in nanoparticle delivery to normal tissues is probably related to the increased bioavailability than to the spillover effect.

The spillover effect is a double-edged sword. The changes in pharmacokinetics of therapeutic nanoparticles affect their toxicity for macrophage-rich organs, which should be assessed in clinical trials. On the other hand, this effect enables more precise targeting of certain organs. For example, depletion of liver and spleen macrophages was used to increase the delivery of bone-targeted nanoparticles to fracture femur with therapeutic effects^103^.

### Safety and toxicity of nanoparticle induced MPS blockade

Macrophages is an ancient and multifunctional type of cells. They play an essential role in the regulation of cell activity, pathogen recognition, and maintaining innate immune response. Compromising of these important functions is one of the issues that should be addressed in this discussion.

The damage of Kupffer cells increases vulnerability of the body to bacterial infection and sepsis^43^. Additionally, the blockade may indirectly hamper adaptive immunity by depleting involved opsonins^104^ or impairing antigen presentation to B cells^105^. To mitigate these potential risks, it is crucial to ensure that macrophage activity is rapidly and fully recovered after the treatment. The depletion of the MPS with toxic substances requires several weeks to repopulate the eliminated macrophages from monocytes^43^. The more sparing recent approaches that employ liposomes and other biocompatible nanoparticles have more transient effect lasting from hours to days^32,69^. After that the phagocytic activity of the MPS bounces back to normal levels. The clearance of fluorescently labelled bacteria does not differ in the control untreated group and the one with the resolved liposomal blockade^32^. On the other hand, the blockade with sensitised red blood cells slows down the clearance of both Gramm-negative and Gramm-positive bacteria only 1.3-1.5-fold, while the circulation of nanoparticles is enhanced by an order of magnitude^69^. The moderate effects of blockade on the bacterial elimination may be explained by involvement of additional innate-immunity mechanisms such as activation of complement and membrane lysis or bacteria uptake with toll-like and pattern recognition receptors.

Another serious concern is potential adverse reactions which may be triggered by blocking nanoparticles and may alternate the antitumour immune response. Blocking nanoparticles interact both with tissue-resident macrophages and blood phagocytes such as monocytes and neutrophils. The macrophages in the liver and spleen participate in the clearance of circulating tumor cells, preventing the formation of metastases. Monocytes migrate to the tumour site and differentiate into the tumour-associated macrophages (TAMs), while neutrophils migrate to the inflammation sites and regulate immunosuppression and angiogenesis. Nanoparticles can modulate the polarisation of immune cells and reprogram their function; however, the direction of polarisation greatly depends on the type of nanomaterial and its properties^106^. M1-like phenotype is preferable for TAMs to exert oncolytic functions by promoting the immune responses^107^. On the other side, M1 macrophages in MPS will faster eliminate the drug-loaded nanoparticles in course of the treatment, compared to M2 macrophages^93^. Cell reprogramming may alter their interaction with tumour cells and this issue should be accessed in further studies of the MPS blockade.

The administration of high doses of nanomaterials may be associated with cardiopulmonary distress and other infusion reactions in susceptible human patients. The mechanism of this phenomenon involves multiple immunological pathways. Inadvertent complement activation by nanoparticles induces pseudoallergy reaction by production of anaphylatoxins, mast cell activation and secondary mediators release. On the other side, nanoparticle interaction with pulmonary intravascular macrophages through Fcγ receptors also initiate downstream signalling pathways with inflammatory mediator release^108^. Severity and probability of the infusion reactions may be decreased by premedication of patients with corticosteroids, prostaglandin inhibitors and antihistamines^109^. Also, slowing down the infusion rate of nanomedicines decreases cardiopulmonary distress in animal models and patients^110^. Monitoring these reactions in clinic is required at first hours after induction of the MPS blockade.

Finally, the long-term deposition of blocking nanomaterials in macrophages may induce oxidative and inflammatory stresses and associated toxicity. Potential adverse effects for liver and spleen should be contemplated carefully, evaluating the safety of the core and the coating materials. In recent studies liposomes and lipid vesicles, polymeric, inorganic nanoparticles and sensitised cells have been used for induction of MPS blockade.

Liposomes and lipid nanoparticles have been so far one of the most clinically successful formulations for drug delivery. They are also one of the most used and effective type of nanoparticles to induce the MPS blockade in recent years, although the exact used lipid composition varies. The lipid-based formulations generally demonstrate a negligible toxicity for the innate immune system. The phosphatidylcholine/cholesterol liposomes induce the MPS blockade in 1 h after the injection, but the MPS fully recovered in 1 day. It did not have any impact on the liver function as measured by the serum alanine transaminase (ALT) blood levels, weight, and behavioural patterns^32^. The liposomes of different 1,2-dipalmitoyl-sn-glycero-3-phosphoglycerol (DPPG)/cholesterol or soy lecithin/cholesterol formulations have not cause any long-term functional or structural changes in organs either despite being effective in inducing the blockade^35^. These notions be also attributed to the particles of the related chemical nature such as exosomes from human blood^23^, intralipid^67^, or albumin-methyl palmitate nanoparticles^52^. Although, it should be noted that methyl palmitate alone has been considered toxic to macrophages^52^. At the same time the safety profile of the lipid-based blocker may dramatically affected and eventually determined by the functional groups on its surface. For instance, the Ni^2+^ functionalised liposomes effectively prolonged the circulation of subsequently administered iron oxide nanoparticles, but induced animals’ death in the experimental group^78^.

The polymer-based nanoformulations used for the MPS blockade in general have similarly moderate toxicity as liposomes. In the recent years acrylamide nanogels, hydroxyethyl starch-grafted-polylactide co-polymer (HES-g-PLA) and gelatine nanoparticles were used for the that purpose. The HES-g-PLA particles did not cause any elevation in ALT or creatine kinase levels compared to control group, neither they drove any morphological changes in major organs. The gelatine nanoparticles were even used in human subjects^81^. However, in high concentration polymer nanoparticles may induce some liver damage as in case of stiff acrylamide-based nanogels. The administration of the nanoparticles increased the ALT levels and white blood cells count compared to controls or to the blockade with softer gels, which might be attributed to the increased liver burden^65^.

Stabilised uncoated inorganic nanoparticles from biocompatible or relatively inert materials have been favoured as blockers starting the very first attempts to investigate the MPS blockade^77^. Nevertheless, even if the short-term toxicity profile of these particles demonstrate safety, they accumulate in tissues and long-term toxic effects should be carefully monitored. For instance, the half-life of iron oxide nanoparticles in mice varies from several weeks to months depending on the coating^111^, whereas gold nanoparticles can be found in organism as long as 1 year after the administration^112^. The major inorganic particles used in the last decades for the MPS blockade were stabilised silicon, iron oxides and gold nanoparticles. Every published study included the systemic toxicity assay by histological or biochemical methods. Despite reported slight elevation in ALT levels immediately after the treatment, in general they did not induce any significant long-term inflammatory, dystrophic, or necrotic changes in major organs when used as a blockers^63,64,80^. However, in short-term iron-based nanoparticles stimulated the growth of macrophage population, which might have been the response to the iron catabolism^111^. Gold nanoparticles even in high doses demonstrated low toxicity, despite a pronounced accumulation in liver sinusoidal cells^80^. However, a slow rate of inorganic nanoparticles degradation may compromise the repeated induction of the blockade if it is required by the therapeutic regimen.

The main toxic concern of the MPS blockade with anti-RBC antibodies attributes to induced anaemia^69^. The MPS blockade with 34-3 C antibody reduces haematocrit from 50% to 45% level within first 4 days after injection, and erythrocytes level restores at day 8 after the treatment. Also, it increases aspartate aminotransferase (AST) and creatinine levels 1 day after induction. While it does not cause any systemic inflammatory response, a substantial increase in the MCP-1 concentration, a chemokine that recruits monocytes, was observed 12 h after antibody administration. It correlates with the increased number of Kupffer cells in the liver and macrophages in spleen 10 days after the treatment. No other changes in major organs or long-term disruption of the MPS was observed. Finally, cytoblockade is the only approach for which multiple induction has been shown with the same toxicity profile. Importantly, the toxicity of the MPS-blockade may depend on the antibody clone used for RBC sensitisation. Another anti-RBC antibody, TER-119, induced severe haemolysis of erythrocytes, with corresponding increase in blood levels of ALT, AST, and bilirubin as well as presence of protein in the urea. The similar intravascular toxicity was observed for several cases of anti-RhD antibody therapy of immune thrombocytopenia in clinic^113^. The selected clones for the MPS blockade should, therefore, avoid causing haemolysis or haemagglutination in patients.

### Perspectives of the MPS blockade

Over the last century, the MPS blockade has progressed from a tool for studying the role of tissue-resident macrophages in the humoral immune response to an adjuvant method for therapeutic gene and drug delivery. Today it has become a powerful approach to boost the circulation time and therapeutic efficacy of nanomedicines. Due to the research efforts of the last two decades its potency has increased by 40%. However, the effect of the blockade on tumour delivery of nanomedicines is currently too moderate for successful clinical translation and the precise mechanism is still being investigated. The MPS blockade is at risk of going on another cycle of negligence and revival. Here we suggest the questions to be addressed to avoid that.

Firstly, in this perspective we describe the combined mechanism of the blockade, which recruits multiple pathways that regulate activity of tissue resident macrophages. The precise input of certain links to this process remains unclear and should be investigated. This knowledge is crucial for fine tuning efficacy, safety, and duration of the blockade.

Secondly, the development of safe and selective blocking agents is a significant problem in the MPS blockade studies. There are two strategies we consider worth pursuing. The first one encompasses comprehensive investigation of the immune, cellular, and molecular mechanisms underlying the blockade. The obtained data would inform the rational design of small molecules or nanoparticle formulations with desired properties. The second strategy involves large-scale screening of already developed safe nanomaterials as the blocking particles for MPS. Such library should include the growing repertoire of biodegradable materials, as well as the set of macromolecular compounds that are be systemically administered without significant adverse effects. Intralipid, which is used for parenteral nutrition, serves as an excellent example in this regard^67^, and other promising materials include artificial blood substitutes and polymers used as plasma volume expanders.

Thirdly, it is crucial to address the regulatory barriers that may impede the translation of MPS blockers to the clinic. We envision that MPS blockers would primarily be utilised as adjuvants to existing therapeutic approaches. From a regulatory standpoint, two potential strategies for their development are to be considered. The first involves the development of a standalone drug that is administered prior to the therapeutic molecule to enhance its effectivity. The blocker would be considered as investigational new drug and will have to undergo a standard sequence of trials to be approved for clinical use. This approach would be rational only if the blocker demonstrates outstanding safety, efficacy, and universality in animals. The second strategy involves repurposing existing compounds that are already on the market and are known to induce the MPS blockade. This approach avoids the need for extensive drug development and regulatory approval processes, as these compounds have already been approved for other indications. One example of a repurposed compound is chloroquine, an antimalarial drug that affects endocytosis and was tested for MPS cells priming^20^. Another example is IVIG (intravenous immunoglobulin), which is used in the treatment of immune thrombocytopenia^114,115^. IVIG disrupts Fc-mediated cell phagocytosis, potentially through blockade of Fc receptors^116^. These drugs, along with several others^117^, modulate the uptake of Kupffer cells and other macrophages in MPS system. They are readily available in market, but additional investigations required to evaluate their safety and efficacy in combination with nanoparticle-based chemotherapy.

Finally, for a better interstudy analysis and, hence, understanding of the blockade mechanisms we suggest implementation of harmonised reporting standards for future research in the field. Based on the meta-analysis, performed here, the following properties are of crucial importance:

1. Size, polydispersity, composition and ζ-potential of the blocking and tracer particles.
2. Dosage regimen for blocking and tracer agents. In the light of the latest studies^80^ besides standard mg/kg, we suggest adding the precise number of the particles per injection.
3. Pharmacokinetics in blood and biodistribution of both blocking and tracer particles. The data on blocking particles will promote understanding an optimal ratio between macrophage-binding and free-circulating particles necessary for the most pronounced effect. The circulation time of tracer particles should be characterised by classic parameters such as t_1/2_ or AUC.
4. Blood pharmacokinetics and biodistribution should be assessed in animals with tumours if study reporting a prospective anti-cancer therapy with the MPS blockade.

In conclusion, MPS blockade is universal approach having potential to enhance the efficiency and accuracy of nanoparticle-based therapies. Safety remains the major issue to be cleared before discussing the future clinical use of this method. Repurposing of the existing drugs which have undergone through the years of pharmacovigilance stands as the fastest and the most promising way to do that.

### Supplementary information

Supplementary Information

Description of Additional Supplementary Files

Supplementary Data 1

Supplementary Data 2

### Source data

Source Data

## Data Availability

The raw data for Figs. 3a–j, 4a–c and Supplementary Figs. 6 and 7 are available in Supplementary Dataset 1, for Fig. 3k in Supplementary Dataset 2. Statistical data are available in the source data file. All data are available from the corresponding authors upon request. Source data are provided with this paper.
