# Supplementary material for: Rediscovery of mononuclear phagocyte system blockade for nanoparticle drug delivery
Source: Nat Commun. 2024 May 22;15:4366. doi: 10.1038/s41467-024-48838-5 (PMC11111695; doi:10.1038/s41467-024-48838-5)
Supplement: Supplementary file 1 — Supplementary Information [file 41467_2024_48838_MOESM1_ESM.pdf]

Supplementary Information

Rediscovery of mononuclear phagocyte system blockade for nanoparticle drug delivery

Ivan V. Zelepukin[1,2#*], Konstantin G. Shevchenko[3#*], Sergey M. Deyev[2]

1 – Department of Medicinal Chemistry, Uppsala University, 751 23 Uppsala, Sweden

2 – Shemyakin-Ovchinnikov Institute of Bioorganic Chemistry of the Russian Academy of Sciences, 117997, Moscow, Russia

3 – Department of Chemistry, Boston University, Boston, Massachusetts 02215, United States

# These authors contributed equally

* Corresponding authors e-mail: ivan.zelepukin@ilk.uu.se (I.V. Zelepukin); ks627@bu.edu (K.G. Shevchenko)

Data collection and standardisation

Papers on MPS blockade strategies were collected by searching Google Scholar and PubMed databases. The starting keywords were “macrophage”, “RES”, “reticuloendothelial system”, “MPS”, “Mononuclear phagocyte system” + “blockade”, “saturation”, “priming”, “preconditioning”. The time range was 1960-2023 (July 1st). We identified and reviewed 153 works and performed the meta-analysis of the studies which employed nanoparticles and cells as blocking agents since they have similar mechanisms of action.

Following data were extracted and summarized in the Supplementary Datasets 1 and 2: properties of the blocking and tracer nanoparticles (composition, size, injected dose), time between injection of blocking and tracer particles, presence of targeting strategy for tracer particles, animal model, presence and type of tumour, blood pharmacokinetics parameters ($t_{1/2}$ increase after the blockade induction, $AUC_{0\text{-}t}$ increase after the blockade induction), tissue biodistribution of the particles (concentration increase in tumour, spleen, liver and lungs after the blockade induction). Supplementary Dataset 1 describes all values for the MPS blockade studies, where the blockade was induced with nanoparticles or cells. Supplementary Dataset 2 describes all the reported studies, in which an improvement in therapeutic efficacy was observed after the blockade induction (tumour inhibition growth or animal survival prolongation). Where possible, the data were gathered directly from the papers, if only the graphs were reported, PlotDigitizer Online app was used to manually collect the values.

For the blood pharmacokinetics analysis from Supplementary Dataset 1, we used half-life time ($t_{1/2}$) ratio of nanoparticles after and before the blockade. If the data were reported in graphical form, the $t_{1/2}$ was calculated from Elimination rate constant (K_{el}) using equation: $t_{1/2} = \ln(2)\,/K_{el}$. If the MPS blockade effect was reported at several time points, data point with maximum efficacy was used for comparison. For Supplementary Dataset 2, we used $AUC_{0\text{-}t}$ ratio values for blood circulation comparison since in most cases the blood pharmacokinetics significantly deviated from monoexponential behaviour.

For biodistribution studies "tumour and tissue delivery increase" shows the ratio of nanoparticle concentration at certain time-point after and before the MPS blockade. Delivery efficacy was analysed by $AUC_{0\text{-}t}$ using trapezoidal model if several data points were reported.

We plotted the results as Tukey-type box graphs, describing median and 25-75% percentiles, whiskers show 1.5-fold interquartile range. We used median values for comparison as it depends less on the variability of data than mean value.

Statistical analysis

In statistical analysis, the outcome variables were "$t_{1/2}$ increase" in blood pharmacokinetics analysis and "tissue delivery increase" in tumour, liver, spleen, and lungs accumulation comparison. Kolmogorov-Smirnov normality test was performed for the outcome variables (Supplementary Table 1). In addition, Quartile-Quartile (Q-Q) plots were plotted for each dataset ($t_{1/2}$ increase – Supplementary Figure 1, tumour, liver, spleen, and lungs delivery increase – Supplementary Figures 2, 3, 4, 5, respectively).

If normality assumption was violated, Box-Cox transformation was performed. The data were transformed according to function F(y):

$$F(y) = \frac{y^{\lambda} - 1}{\lambda}$$

The optimized λ values were: (-0.5) for $t_{1/2}$ increase; (-1.2) for tumour delivery increase; (-1.2) for liver delivery increase; (0.4) for spleen delivery increase; (-0.2) for lungs delivery increase. After the Box-Cox transformation, Kolmogorov-Smirnov normality test shows normal distribution of transformed variables (Supplementary Table 1), as well as Q-Q plots of F(y) have linear dependence (Supplementary Figures 1-5). Hence, for statistical comparison, the analysis of variance (ANOVA) with Tukey's post-hoc test was applied for p values calculation. p value less than 0.05 was determined statistically significant. Supplementary Tables 2-5 show descriptive statistics, as well as p-values for the analysed data.

Supplementary Table 1. Results of Kolmogorov-Smirnov normality test before and after Box-Cox data transformation

Group	p-value	
$t_{1/2}$ increase	<0.0001	Reject normality
$t_{1/2}$ increase, transformed	1	Can't reject normality
Tumor delivery increase	<0.0001	Reject normality
Tumour delivery increase, transformed	0.68	Can't reject normality
Liver delivery increase	<0.0001	Reject normality
Liver delivery increase, transformed	0.71	Can't reject normality
Spleen delivery increase	0.32	Can't reject normality
Lungs delivery increase	0.0001	Reject normality
Lungs delivery increase, transformed	0.15	Can't reject normality

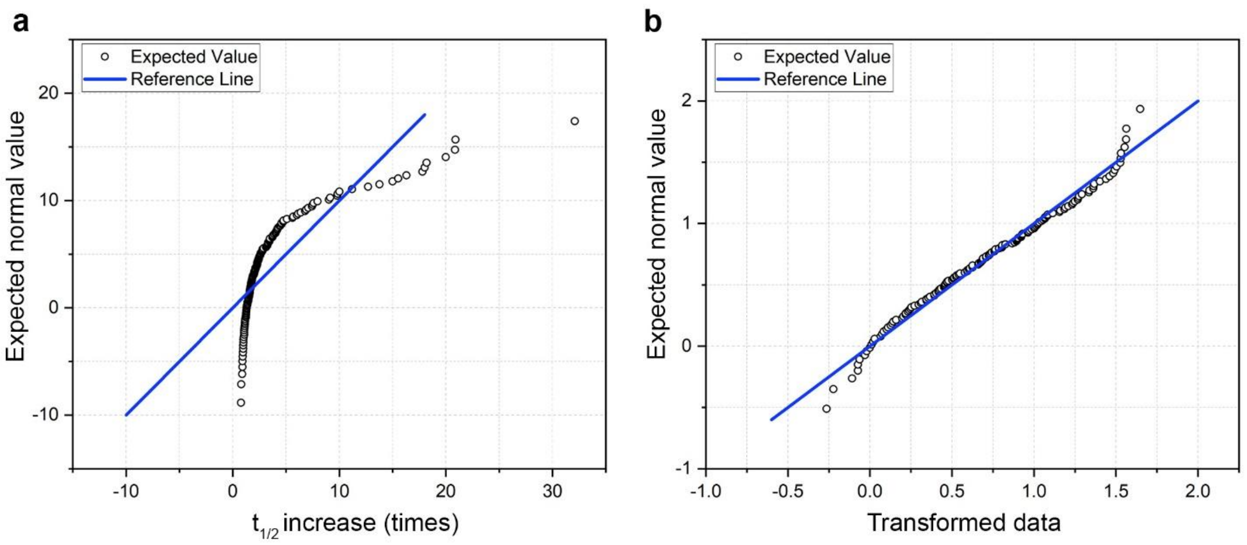

Supplementary Figure 1. Quantile–Quantile plot of "$t_{1/2}$ increase" data before **(a)** and after **(b)** Box-Cox transformation. Reference lines data: **(a)** $\mu = 4.273$, $\sigma = 4.997$; **(b)** $\mu = 0.702$, $\sigma = 0.456$.

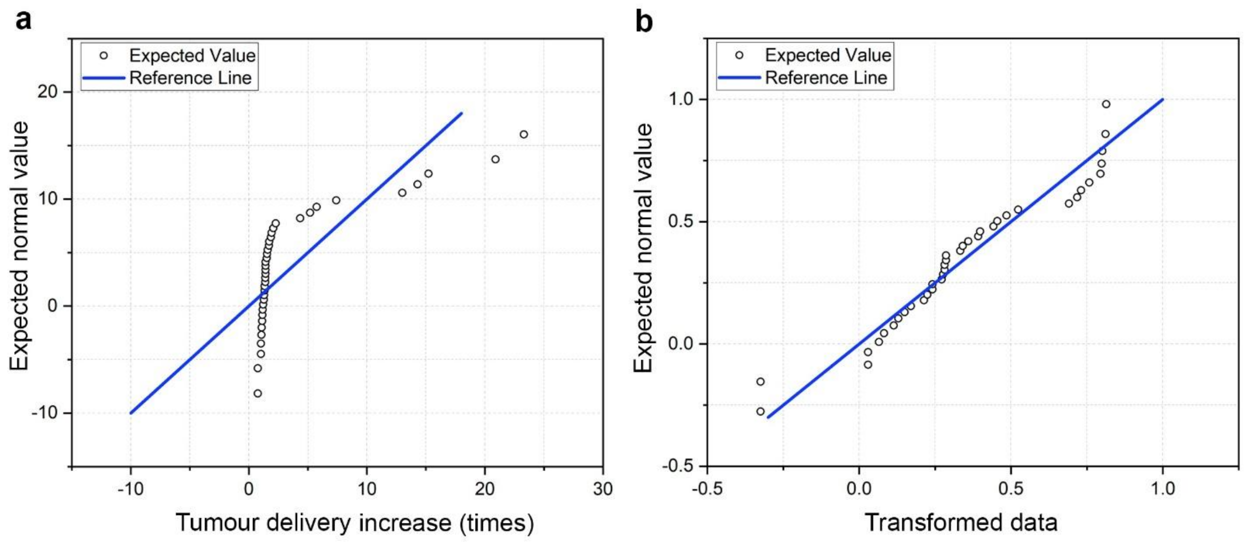

Supplementary Figure 2. Quantile–Quantile plot of "tumour delivery increase" data before **(a)** and after **(b)** Box-Cox transformation. Reference lines data: **(a)** $\mu = 3.942$, $\sigma = 5.667$; **(b)** $\mu = 0.352$, $\sigma = 0.294$.

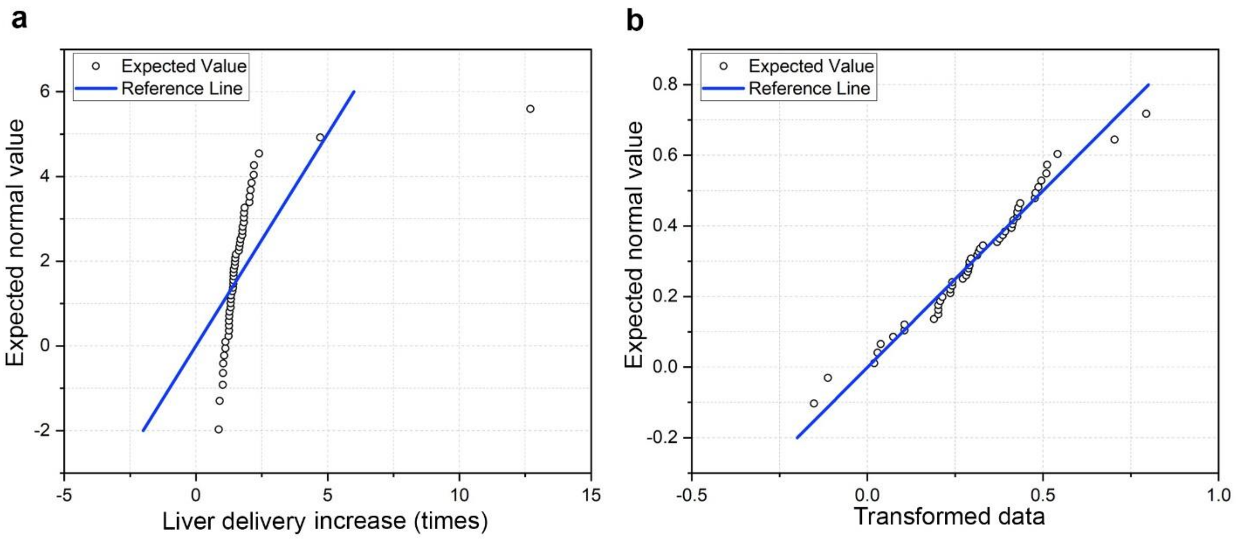

Supplementary Figure 3. Quantile–Quantile plot of “liver delivery increase” data before **(a)** and after **(b)** Box-Cox transformation. Reference lines data: **(a)** $\mu = 1.812$, $\sigma = 1.692$; **(b)** $\mu = 0.307$, $\sigma = 0.184$.

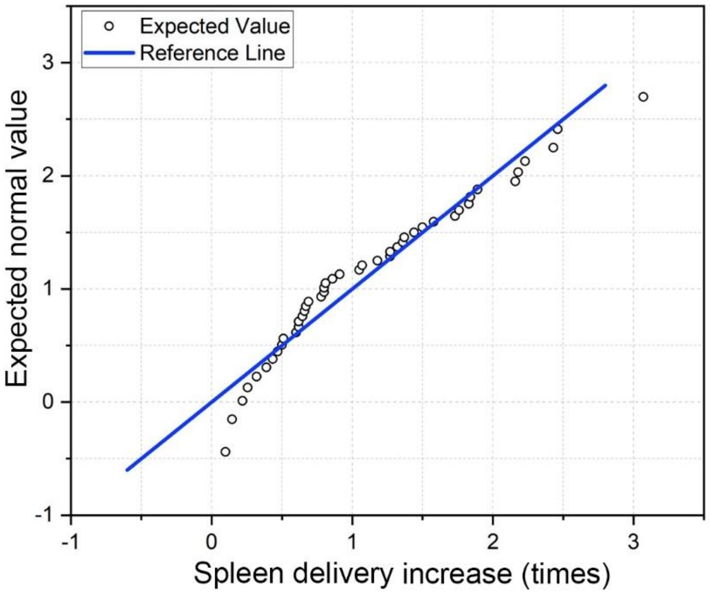

Supplementary Figure 4. Quantile–Quantile plot of “spleen delivery increase”. Reference lines data: $\mu = 1.129$, $\sigma = 0.712$.

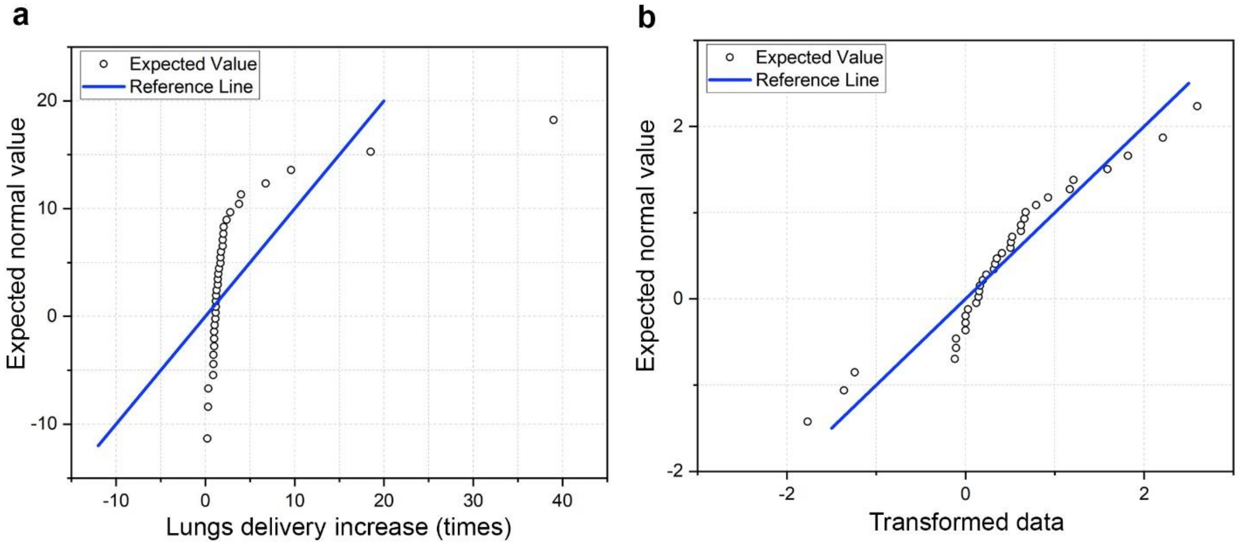

Supplementary Figure 5. Quantile–Quantile plot of "lungs delivery increase" data before **(a)** and after **(b)** Box-Cox transformation. Reference lines data: **(a)** μ = 3.44, σ = 7.03; **(b)** μ = 0.405, σ = 0.869.

Supplementary Table 2. Descriptive statistics of Supplementary Dataset 1 for "$t_{1/2}$ increase" variables. Bold ANOVA values show statistically significant difference ($p < 0.05$).

**Variable**	**Level**	**n**	**Mean**	**Median (Q2)**	**Q1**	**Q3**	**ANOVA**
All studies		144	4.27	2.33	1.49	4.45	
Year	Before 2000	93	4.13	2.02	1.29	4.27	**0.027**
	After 2000	51	4.53	2.80	1.84	5.10	
Animal	Human	10	2.79	2.43	1.46	3.80	0.749
	Mice	70	3.73	2.33	1.60	4.11	
	Rats	57	5.28	2.30	1.34	7.43	
Type of tracer and blocking particles	Similar	49	4.63	3.20	1.88	5.10	**0.019**
	Non-similar	95	4.09	2.05	1.30	4.01	
Type of blocking particles	Organic	79	3.49	2.24	1.48	4.05	0.418
	Inorganic	65	5.23	2.50	1.50	6.02	
Size of blocking particles	<200 nm	36	3.26	2.18	1.48	3.84	**0.028**
	>200 nm	35	5.13	3.44	2.07	6.40	
Type of tracer particles	Short-circulating	73	6.09	3.5	1.6	7.6	**0.035**
	Long-circulating	13	1.97	1.68	1.55	2.33	
Time after blocking particle injection	<1 h	31	2.37	1.78	1.38	3.50	0.138
	1 h	72	4.62	2.09	1.17	4.76	
	>1 h, <2 h	21	2.39	1.80	1.15	2.41	
	>2 h	38	2.17	1.61	1.12	2.33	
Immunodeficiency	Immunodeficient, with tumour	5	2.16	1.68	1.67	1.81	0.197
	Normal, with tumour	6	2.81	2.94	1.24	3.50	
	Normal, without tumour	128	4.44	2.32	1.47	4.60	

Supplementary Table 3. Descriptive statistics of Supplementary Dataset 1 for "tumour delivery increase" variables. Bold ANOVA values show statistically significant difference ($p < 0.05$).

**Variable**	**Level**	**n**	**Mean**	**Median (Q2)**	**Q1**	**Q3**	**ANOVA**
All studies		38	3.94	1.42	1.21	2.28	
Tumour type	Allografts	22	4.47	1.63	1.18	4.36	0.477

	Xenografts	16	3.21	1.36	1.25	1.77	
Targeting of tracer particles	Targeted	16	11.47	10.20	5.20	18.07	**< 0.0001**
	Non-targeted	27	1.38	1.38	1.15	1.55	

Supplementary Table 4. Descriptive statistics of Supplementary Dataset 1 for "tissue delivery increase" variables. Bold ANOVA values show statistically significant difference ($p < 0.05$).

**Variable**	**Level**	**n**	**Mean**	**Median (Q2)**	**Q1**	**Q3**	**ANOVA**
Liver accumulation increase	All studies	49	1.81	1.44	1.27	1.82	
	Size < 200 nm	15	1.37	1.33	1.24	1.43	0.517
	Size > 200 nm	19	1.46	1.43	1.27	1.68	
Spleen accumulation increase	All studies	45	1.13	0.91	0.62	1.58	
	Size < 200 nm	14	1.58	1.60	0.91	2.18	**0.029**
	Size > 200 nm	16	0.93	0.75	0.50	1.45	
Lungs accumulation increase	All studies	35	3.44	1.41	1	2.06	
	Size < 200 nm	7	1.67	1.17	1	2.03	0.580
	Size > 200 nm	15	6.07	1.44	1.03	6.77	

Supplementary Table 5. Descriptive statistics for Supplementary Dataset 2.

**Group**	**n**	**Mean**	**Median (Q2)**	**Q1**	**Q3**
Blood AUC increase	8	2.58	2.37	1.59	3.15
Tumour delivery increase	7	1.75	1.78	1.6	1.9

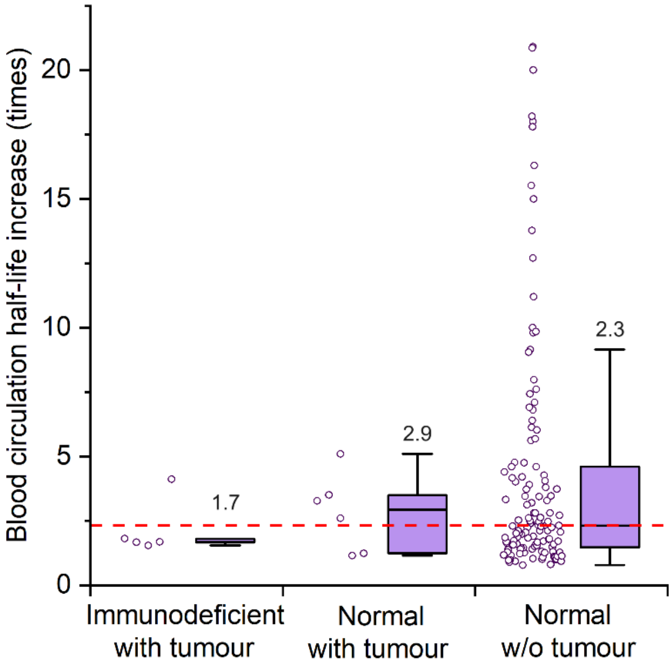

Supplementary Figure 6. The increase in half-life time of tracer nanoparticles after the induction of the MPS blockade in animals with different immune status. Red line indicates median efficiency derived from all data sets and equals to 1.4-fold increase. The boxes represent median, the 25th to 75th percentiles and the whiskers show 1.5 interquartile range. Analysed data points are plotted left to the boxplots. Median values are reported on the graph.

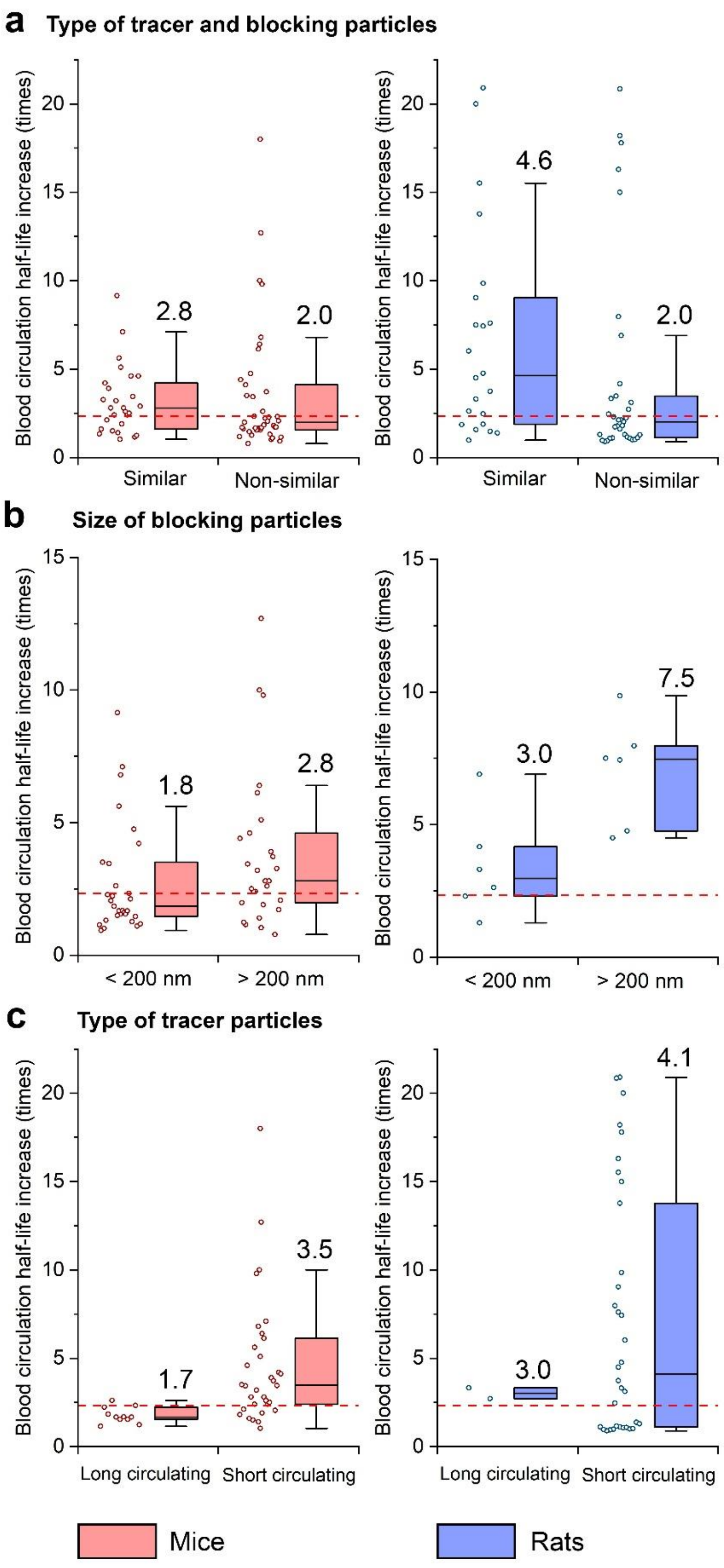

Supplementary Figure 7. (a-c) The increase in half-life time of tracer nanoparticles after the induction of the MPS blockade in mice (left, red) and rats (right, blue). Red line indicates median efficiency derived from all data sets and equals to 2.3-fold increase. The boxes represent median, the 25th to 75th percentiles, and the whiskers show 1.5 interquartile range. Analysed data points are plotted left to the boxplots. Median values are reported on the graphs.
