# Supplementary material for: Rediscovery of mononuclear phagocyte system blockade for nanoparticle drug delivery
Source: Nat Commun. 2024 May 22;15:4366. doi: 10.1038/s41467-024-48838-5 (PMC11111695; doi:10.1038/s41467-024-48838-5)
Supplement: Supplementary file 2 — Description of Additional Supplementary Files [file 41467_2024_48838_MOESM2_ESM.pdf]

Description of Additional Supplementary Files

File Name: Supplementary Data 1
Description: Extracted and summarized data on properties of the blocking and tracer nanoparticles (composition, size, injected dose), time between injection of blocking and tracer particles, presence of targeting strategy for tracer particles, animal model, presence and type of tumour model, blood pharmacokinetics parameters ($t_{1/2}$ increase after the blockade induction, $AUC_{0\text{-}t}$ increase after the blockade induction), tissue biodistribution of the particles (concentration increase in tumour, spleen, liver and lungs after the blockade induction). The dataset encompasses all MPS blockade studies, where it was induced with nanoparticles or cells.

File Name: Supplementary Data 2
Description: Extracted and summarized data on properties of the blocking and tracer nanoparticles (composition, size, injected dose), blood pharmacokinetics parameters ($AUC_{0\text{-}t}$ increase after the blockade induction), type of tumour model, and tumour delivery of the therapeutic tracer particles. The dataset encompasses all reported studies, where an improvement of therapeutic efficacy was observed after the blockade induction (tumour inhibition growth or animal survival prolongation).
